# Cardiac imaging with ^18^F-fluorodeoxyglucose PET/MRI in hypertrophic cardiomyopathy

**DOI:** 10.1007/s12350-016-0686-x

**Published:** 2016-10-14

**Authors:** Yasuchika Takeishi, Atsuro Masuda, Hitoshi Kubo, Hideyuki Tominaga, Noboru Oriuchi, Seiichi Takenoshita

**Affiliations:** 1 Department of Cardiovascular Medicine, Fukushima Medical University, 1 Hikarigaoka, Fukushima, 960-1295 Japan; 2 Advanced Clinical Research Center, Fukushima Medical University, Fukushima, Japan

## Introduction

Simultaneous imaging with magnetic resonance imaging (MRI) and positron emission tomography (PET) is valuable in various cardiac diseases.^1^,^2^ Altered myocardial metabolism has been reported in hypertrophic cardiomyopathy (HCM).^3^ In this study, we report an instructive case of HCM with intense ^18^F-fluorodeoxyglucose (FDG) uptake in the hypertrophied myocardium detected by PET/MRI.

## Case Summary

**Figure 1:**
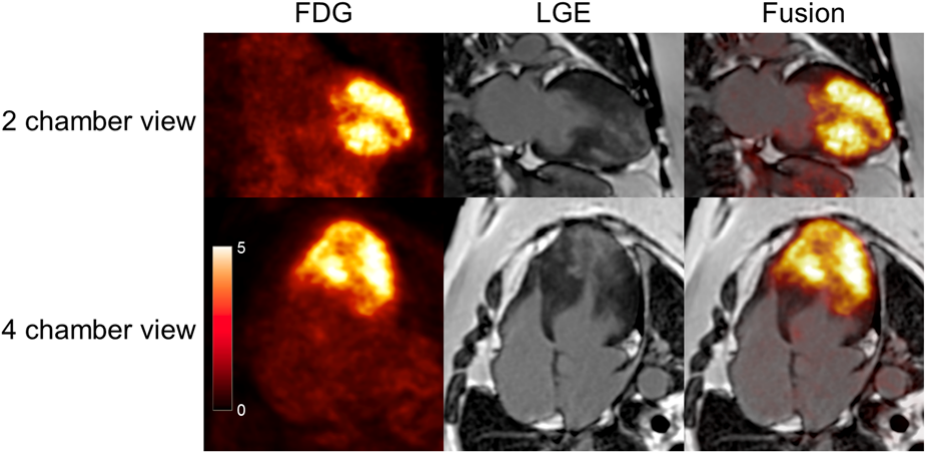
^18^F-fluorodeoxyglucose PET/MRI images of 2- and 4-chamber views. Intense ^18^F-fluorodeoxyglucose (FDG) uptake was observed on the middle walls in the left ventricular myocardium. Late gadolinium enhancement (LGE) was detected on the apex. Fusion images clearly showed the markedly increased FDG uptake in hypertrophied myocardial regions

**Figure 2:**
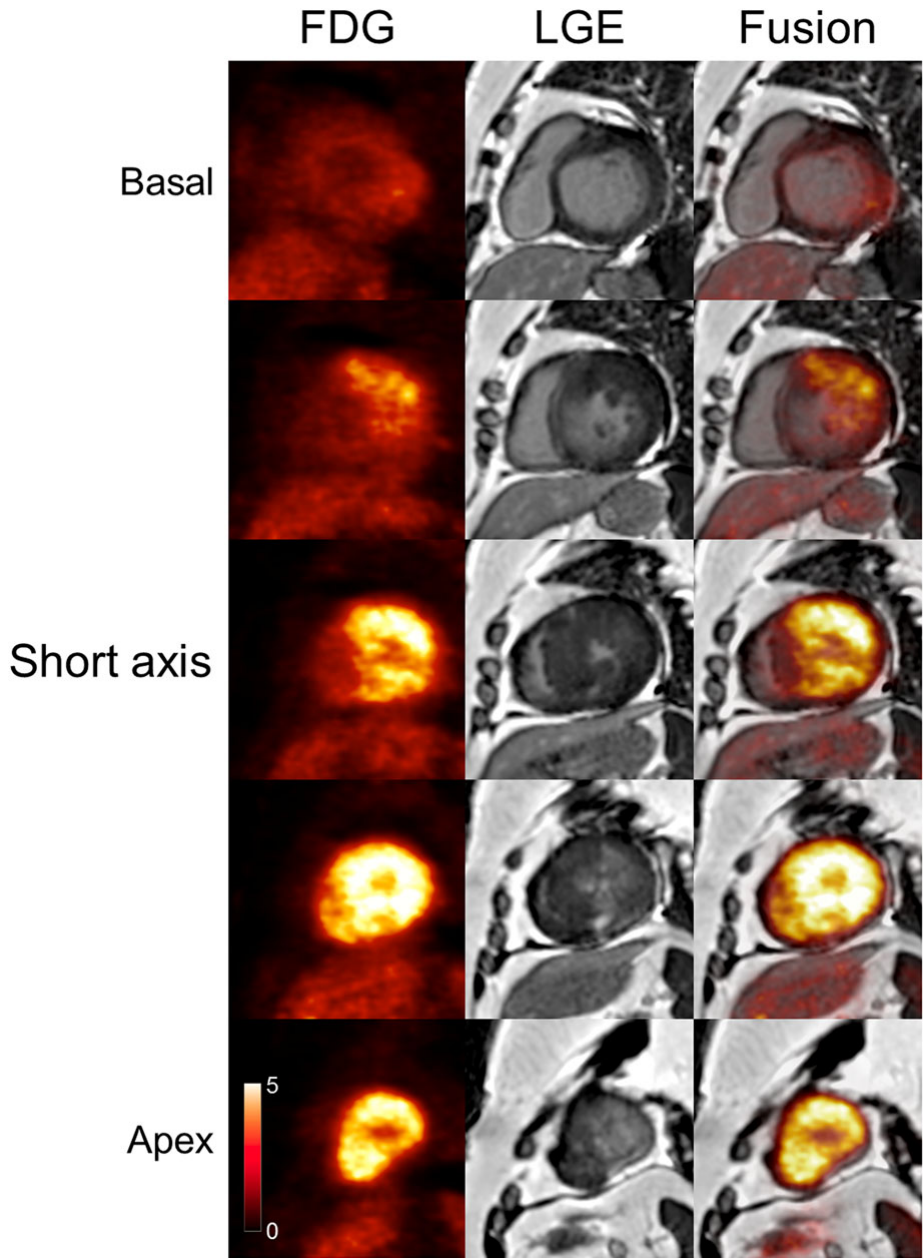
Serial short-axis images of ^18^F-fluorodeoxyglucose PET/MRI. ^18^F-fluorodeoxyglucose (FDG), late gadolinium enhancement (LGE), and fusion images from base (*upper*) to apex (*lower*)

A 57-year-old female was referred to our hospital for severe left ventricular hypertrophy (LVH). Although she had no symptoms and no history of hypertension, echocardiography demonstrated severe LVH in the mid LV to the apex with no asymmetric septal hypertrophy. Thus, we suspected apical type of HCM and performed FDG-PET/MRI. After fasting for more than 18 hours, the patient was administered unfractionated heparin 50 IU/kg intravenously 15 minutes before FDG administration to suppress physiological FDG uptake on the myocardium.^2^ Image acquisition was initiated 60 min after FDG administration. Cine MRI images (Videos 1 and 2 in supplementary material) showed severe hypertrophy in the middle region of the LV with no LV outflow obstruction. However, myocardial thinning and reduced wall motion were observed in the apex. Intense FDG uptake was observed in the LV, suggesting a metabolic substrate switching from fatty acids to glucose, and FDG uptake was relatively low, and late gadolinium enhancement was evident in the apex (Figures 1, 2). Fusion images clearly showed FDG uptake on the hypertrophied myocardium. It should be noted that, although we used prolonged fasting and intravenous heparin, distinguishing physiological uptake of FDG from pathological uptake related to metabolic switching might be sometimes challenging. Simultaneous PET/MRI was able to reconstruct accurate fusion images and provided anatomic and metabolic characterizations of the hypertrophied heart. Myocardial biopsy revealed cardiomyocyte hypertrophy with mere disarray, slight fibrosis, and no inflammatory cell infiltration, consistent with pathological findings of HCM. There were no signs suggestive of secondary cardiomyopathy, which represents cardiac hypertrophy. The PET/MRI is a novel useful imaging technology to evaluate structural and functional abnormalities in HCM.

## Electronic supplementary material

Below is the link to the electronic supplementary material.

Cine MRI of 2-chamber view. Severe myocardial hypertrophy was observed in the left ventricle with the exception of base and apex. Myocardial thinning and akinetic wall motion were detected in the apex. Supplementary material 1 (WMV 177 kb)

Cine MRI of 4-chamber view. Marked left ventricular hypertrophy in the middle region, and myocardial thinning and dysfunction in the apex. Right ventricular function was normal with no right ventricular hypertrophy. Supplementary material 2 (WMV 146 kb)
